# *PAPPA2* Promote the Proliferation of Dermal Papilla Cells in Hu Sheep (*Ovis aries*) by Regulating *IGFBP5*

**DOI:** 10.3390/genes12101490

**Published:** 2021-09-24

**Authors:** Tianyi Wu, Shanhe Wang, Qiunan Jin, Xiaoyang Lv, Wei Sun

**Affiliations:** 1 College of Animal Science and Technology, Yangzhou University, Yangzhou 225009, China; 18362822698@163.com (T.W.); shanhe12315@163.com (S.W.); jqn1006@163.com (Q.J.); dx120170085@yzu.edu.cn (X.L.); 2 Joint International Research Laboratory of Agriculture and Agri-Product Safety of Ministry of Education of China, Yangzhou University, Yangzhou 225009, China

**Keywords:** pregnancy-associated plasma protein A2, insulin-like growth factor binding protein-5, dermal papilla cell, curved hair, Hu sheep

## Abstract

Hu sheep (*Ovis aries*) is a rare white sheep breed, with four different types of lambskin patterns that have different values. However, the genetic mechanisms underlying different types of pattern formation remains unclear. This research aimed to characterize the molecular mechanism of differentially expressed gene *PAPPA2* affecting the pattern type of Hu sheep’s lambskin at the cellular level. Thus, RT-qPCR, EdU and Cell Cycle detection were used to explore the effect of *PAPPA2* and *IGFBP5* (a protein that can be hydrolyzed by PAPPA2) on the proliferation of dermal papilla cells (DPCs) after overexpression or interference with *PAPPA2* and *IGFBP5*. The expression level of *PAPPA2* in straight DPCs was 4.79 ± 1.84 times higher than curved. Overexpression of *PAPPA2* promoted the proliferation of DPCs and also increased the expression of *IGFBP5*. Conversely, overexpression of *IGFBP5* reduced the proliferation of DPCs. However, the proliferation of DPCs was restored by co-overexpression of *PAPPA2* and *IGFBP5* compared with overexpression of *IGFBP5* alone. Thus, *PAPPA2* can affect the proliferation of DPCs through regulating *IGFBP5* and then participate in lambskin pattern determination. Overall, we preliminarily clarified the critical role played by *PAPPA2* during the formation of different pattern in Hu sheep lambskin.

## 1. Introduction

**Figure 1:**
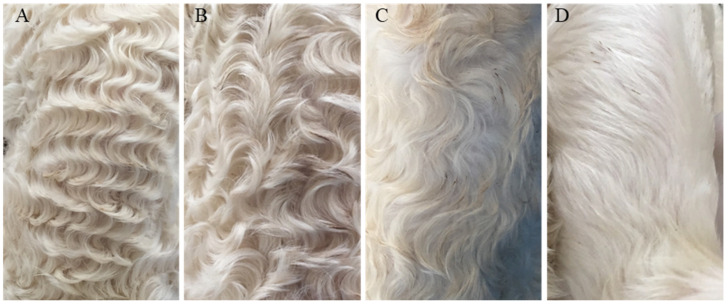
Different lambskin pattern types of Hu sheep. Note: (**A**): Extremely curved. (**B**): Medium curved. (**C**): Little curved. (**D**): Straight.

Hu sheep, native to China, is a rare white lambskin breed, its lambskin has four pattern types: extremely curved (named “Curved” in this article), medium curved, little curved and straight [1] (named “Straight” in this article) (Figure 1). The formation of different pattern types is believed to be related to the length of fetal hair; at the beginning (110 d fetal age), the fetal hair just starts to grow. All hairs are short and straight without bending. When the embryos continues to grow to 120d fetal age, a wave pattern will form with proper hair length, but as the embryos continue to grow (140 d fetal age), the wave pattern in some of the embryos will gradually disappear and change to straight hair because of the excessive length of the fetal hair [2].

The growth of wool is closely related to the development of the hair follicle (HF). The HF is an important accessory organ of the skin, and its main components include the dermal sheath (DS), the outer root sheath (ORS), the companion layer (CL), the inner root sheath (IRS), the matrix (Mx) and the dermal papilla (DP) [3,4,5]. Among them, DP plays an important role in the regulation of both hair follicle development and cycle [6].

Hair growth is associated with a number of factors, among which the regulation of the entire hair follicle by the dermal papilla cells (DPCs), a class of dermal origin cells composed of specialized fibroblasts of mesenchymal origin located in the center of the hair bulb, is highly important. In the early stages of hair development, after the “first dermal signal” induces the formation of the epidermal substrate, the dermal cells are stimulated by the “epithelial signal” from the substrate to agglomerate together and gradually form DP, which take over the hair growth and cycle regulation [7]. DPCs have a powerful ability to induce hair follicle formation, and it has been confirmed that subcutaneous implantation of DPCs alone can form intact hair follicles and generate hairs [6]. Additionally, if the DP of growing hair follicle is removed, the growth of the hair follicle will stop [8].

DPCs express *Wnts*, *R-spondins*, *FGF* and *Noggin* exocrine signaling factors in vivo, all of which promote hair follicle growth and help initiate follicle regeneration [9,10]. For example, it has been indicated that signals from DPCs can regulate the transition of hair follicles from telogen to regeneration [11]. Hair follicles lacking *β-catenin* in DPCs entering catagen earlier, which means that the activity of DPCs regulates the anagen-to-catagen transition [12]. Alkaline phosphatase (*ALP*) as a marker of DPCs was found to reach its highest level in the early anagen phase and gradually decrease in the middle and late anagen phase, which suggested an important role of *ALP* in the anagen phase of hair follicles [13]. *Versican* is characteristically highly expressed in DPCs of anagen hair follicle while progressively decreased in catagen, and is not expressed in telogen DPCs [14]. The expression of insulin-like growth factor binding protein-3 (*IGFBP3*) and insulin-like growth factor binding protein-5 (*IGFBP5*) is significantly increased in DPCs at the end of the hair follicle growth phase [15]. All of the above studies indicate the important role of DPCs in the regulation of the hair follicle cycle.

*IGFs* and their binding proteins have been proved to affect hair curvature. Researchers overexpressed *IGF1* in the inner root sheath and hair medulla of mice then found that it inhibited hair shaft curvature while overexpression of *IGFBP3* partially counteracted the loss of zigzag hairs caused by overexpression of *IGF1* [16]. In the same way, *IGFBP5* is a negative regulator of cell proliferation in hair follicles. It has been found that *IGFBP5* is highly expressed in human curly hair phenotypes, and staining showed that *IGFBP5* has a higher asymmetrical expression on the convex side of ORS of curly hair contrary to the symmetrical *IGFBP5* expression observed in straight hair. Further studies in vitro hair follicle organs revealed that *IGFBP5* has significant curvature-causing effects on human hair morphology [17].

In our previous sequencing study (unpublished data), we identified the gene pregnancy-associated plasma protein-A2 (*PAPPA2*), which is differentially expressed between curved and straight lambskin pattern types of DPCs. This gene is an active metalloprotease in humans, which specifically cleaves IGFBP5 protein and thus regulates cellular utilization of IGF [18]. *PAPPA2* has been shown to affect childhood development [19] as well as skeletal development [20], but fewer studies have been reported on its association with hair growth.

Downregulation of genes is a powerful way to investigate functional involvement in various processes. Small-interfering ribonucleic acids (siRNAs), found by Hamilton [21] firstly, is an important molecular tool which is often used to reduce gene-, and subsequent protein-expression. In 2001, Hutvágner discovered that synthetic siRNAs can induce RNA interference in mammals [22]. Since then siRNA has been widely used to interfere with specific genes in biological research. In this study, we also chosen to use siRNA to interfere with *PAPPA2.*

The goal of this study is to verify the proliferation regulation of *PAPPA2* on Hu sheep DPCs by detecting changes in the cell cycle and cell proliferation of DPCs after altering the expression of *PAPPA2* and *IGFBP5* in Hu sheep DPCs. This result can provide a reference for studying the regulatory mechanism of hair growth in animals, also provide a new direction for the study of the formation mechanism of lamb skin pattern in Hu sheep.

## 2. Materials and Methods

### 2.1. Dermal Papilla Cells Culture

All DPCs used in this experiment were isolated and stored in our laboratory before.

The main procedure was as follows: Step1: we selected healthy two-day-old Hu sheep, sampled a small piece of skin tissue at the left scapula after anesthetizing and treated the wound promptly; Step2: we used microdissection to isolate dermal papilla tissue in the hair follicle; Step3: we used collagenase IV (Solarbio, Beijing, China) to digested and isolated DPCs and used serum-containing medium (FBS came from Gibco, Grand Island, NY, USA) to terminate the digestion; Step4: we centrifugated cell suspensions to collect DPCs then started a DPC primary culture; Step5: we twice completed purification cultures of DPCs and identified the expression of *VIM* (sc-6260, Santa Cruz, Dallas, TX, USA) and *α-SMA* (D221592, bbi-lifesciences, Shanghai, China) using immunofluorescence to verify the cell types.

DPCs were cultured in DMEM/F12 supplemented with 10% FBS and 1% penicillin streptomycin (All from Gibco, Grand Island, NY, USA) at 37 °C with 5% CO_2_. When the cell reaches the logarithmic growth stage, they were seeded in different culture plates according to the experimental design before transfection.

### 2.2. The Extraction of Total Cellular RNA and Synthesis of cDNA

All the following operations are carried out in clean bench, also the centrifuge tubes and other items that used in operations are all RNase-free treated. In order to extract cellular RNA, DPCs were cultured adherently in six-well plates. When cells reached 80–90% confluency, or 24 h after completion of cell transfection, RNA was extracted using RNA simple Total RNA Kit (Tiangen, Beijing, China) according to its instructions.

The cDNA prepared for RT-qPCR was synthesized by using RNA First-Strand cDNA Synthesis Kit (Tiangen, Beijing, China) and the operation steps referred to the product manual. We used 1500 ng total RNA for 20 μL system and stored it at −20 °C after the reverse transcription was completed.

The cDNA prepared for the full-length high-fidelity amplification of the target gene was synthesized by TIANScript II cDNA First Strand Synthesis Kit (Tiangen, Beijing, China) and the operation steps referred to the product manual. Each 20 μL system uses 1000 ng total RNA, 2 μL Oligo-dT (10 μM) and the cDNA was stored at −20 °C after the reverse transcription was completed.

### 2.3. Real-Time Quantitative Polymerase Chain Reaction (RT-PCR)

#### 2.3.1. Primer Design

According to the sheep (*Ovis aries*) gene sequence published by Gene Bank, quantitative primers for mRNA such as *PAPPA2* (Table 1) was designed using NCBI online software (https://www.ncbi.nlm.nih.gov/tools/primer-blast/ (accessed on 9 October 2020)). Tsingke Biotechnology (Beijing) Co., Ltd. is responsible for the synthesis of the primers.

#### 2.3.2. Operation Steps

The experiment was carried out according to the user manual of TB Green™ Premix EX Taq™ II (Takara, Kusatsu, Shiga, Japan), the main steps are as follows:

Prepare the PCR mixture in Real-time PCR reaction plates designed specifically for the qPCR instrument used, this step should be done on ice. Each well includes: 2 × TB Green Premix Ex Taq II: 12.5 μL, PCR Forward Primer(F): 1 μL, PCR Reverse Primer(R): 1 μL, cDNA: 1 μL, RNase-free water: 9.5 μL. Technical repetitions was set 3 or more for each condition. Specifically, the results of “the relative expression of *PAPPA2* mRNA in straight and curved DPCs” and “the relative expression of *IGFBP5* and *PAPPA2* mRNA after transfection of pIGFBP5-O, pIGFBP5-O+pPAPPA2-O and pcDNA3.1 in DPCs” had 4 technical repetitions. Other results of RT-qPCR used 3 technical repetitions. After mixing the reagents thoroughly and clearing the bubbles, CFX96 Connect™ Real-Time System (BIO-RAD, CA, USA) was used for detection.

Protocol: STEP1: 95 °C, 30 s, STEP2: 95 °C, 5 s, STEP3: 60 °C, 10 s, STEP4: 72 °C, 30 s, STEP5: GOTO STEP2 39 times (40 Cycles), STEP6: 95 °C, 10 s, STEP7: Melt Curve 65 °C to 95 °C.

Finally, the 2^−ΔΔCt^ method [23] was used to analyze the experimental data in the Microsoft Excel (Version: 2013, Microsoft, Redmond, WA, USA) with *GAPDH* as the internal reference gene.

### 2.4. Construction of PAPPA2 Overexpression Vector

#### 2.4.1. Primer Design

According to the CDS full-length sequence in the predicted mRNA sequence of the *PAPPA2* gene of sheep provided by NCBI (5382 bp, GenBank Accession: XM_004013823.4), PCR amplification primers were designed using Primer Premier 5 (Version: 5.00, PREMIER Biosoft International, CA, USA). The primer sequence is:

F: AGACCCAAGCTGGCTAGTTAAGCTTGCCACCatgatgtgcttcaagattctgaggg

R: TCGAAGGGCCCTCTAGACTCGAGttactggttttcttcagccctgg

The lowercase is the CDS full-length amplified sequence of *PAPPA2*. The uppercase in the forward primer(F) is the Kozak sequence and the uppercase underlined is the homology arm that is the same as the sequence of the vector *Hin*d III and *Xho* I restriction site region.

#### 2.4.2. Full-Length High-Fidelity Amplification of the PAPPA2 CDS Region

Use PrimeSTAR Max DNA Polymerase reagent (Takara, Kusatsu, Shiga, Japan) for high-fidelity amplification of the full-length PAPPA2 CDS sequence. The reaction system is as follows: PrimeSTAR Max Premix (2×) 25 μL, Forward Primer(F): 1 μL, Reverse Primer(R): 1 μL, cDNA 4 μL, RNase-free ddH_2_O: 19 μL.

Protocol: Denaturation at 98 °C for 10 s, Gradient annealing at 56 °C to 60 °C for 5 s, Extension at 72 °C for 3 min. There are 3 steps and 40 cycles in all. The specificity of the amplified products was checked by 1% agarose gel electrophoresis and MiniBEST Agarose Gel DNA Extraction Kit (Takara, Kusatsu, Shiga, Japan) was used to recover and purify at least 300 μL of the amplified product according to the Kit’s instructions. At last, a micro-ultraviolet spectrophotometer (Life Real, Hangzhou, China) was used to measure the DNA concentration then the purified product was stored at −20 °C.

#### 2.4.3. Restriction of pcDNA3.1 Plasmid

**Figure 2:**
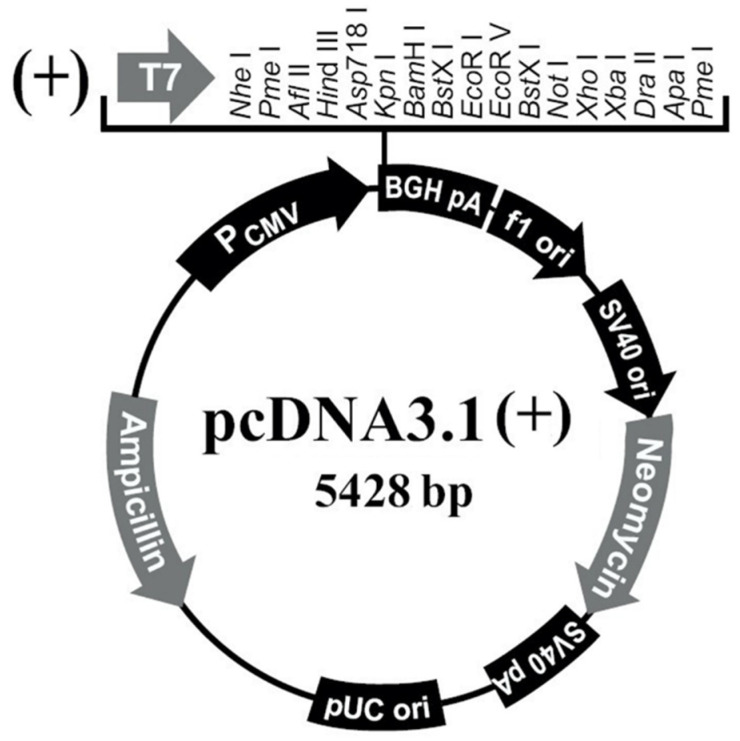
pcDNA3.1(+) illustration.

The pcDNA3.1(+) plasmid (Figure 2) (Youbio, Changsha, China) was digested with restriction enzymes *Hin*d III and *Xho* I (Takara, Kusatsu, Shiga, Japan), and digested at 37 °C for 30 min. The digestion system is shown in Table 2. MiniBEST Agarose Gel DNA Extraction Kit (Takara, Kusatsu, Shiga, Japan) was used to digest the restricted product to obtain linear *Hin*d III-*Xho* I CUT pcDNA3.1(+) vector. Finally, we measured the concentration and store the vector at −20 °C.

#### 2.4.4. Ligation of PAPPA2 Fragment with Linear Vector

During the ligation process, the manual of Trelief™ SoSoo Cloning Kit (Tsingke, Beijing, China) was referred to. Before ligation, the dosages were calculated respectively according to the concentration of the linear vector and insert fragment, the rate is 1 (linear vector):10 (insert fragment). The final ligation system is: 2×SoSoo Mix 5 µL, linear vector 0.5 µL, *PAPPA2* target fragment 4.5 µL. The ligation process continued at 50 °C for 45 min.

The next step was to transform the ligation product into Trelief™ 5α Chemically competent cells (Tsingke, Beijing, China) according to the instructions.

After the competent has resuscitated, 100 µL of bacterial solution was evenly spread on the solid medium supplemented with ampicillin with a spreader, and the petri dish was sealed with a parafilm and placed in a constant temperature incubator at 37 °C overnight. After overnight incubation, a typical colony away from the satellite-shaped bacteria was identified in the petri dish, and the colony was placed in a tube containing 10 mL ampicillin-resistant LB broth. The tube should shake for 16 h.

2 mL of the bacterial solution that appears cloudy and turbid after overnight culture was took then extracted for plasmids using TIANprep Rapid Mini Plasmid Kit (Tiangen, Beijing, China) according to its instructions. The plasmids then will be examined by double digestion verification using the double enzyme digestion system mentioned above.

The plasmid which has been successfully constructed was sent to Beijing Tsingke Biotechnology Co., Ltd. (Nanjing, China) for sequencing and comparison. At the same time, we used EndoFree Mini Plasmid Kit II (Tiangen, Beijing, China) to extract the plasmid in the remaining 8 mL bacterial solution which would then be used for cell transfection according to its instructions. The successfully constructed vector was named pPAPPA2-O.

#### 2.4.5. Construction of IGFBP5 Overexpression Vector

The *IGFBP5* vector is synthesized by Generay Bioengineering Co., Ltd. (Shanghai, China) The target fragment sequence referred to the *IGFBP5* mRNA sequence (NM_001129733.1) registered on NCBI (https://www.ncbi.nlm.nih.gov/ (accessed on 10 January 2021)). The CDS region of the gene is 816 bp long, Kozak sequence was added to the 5′ end of the CDS region as well.

The successfully constructed plasmid was sent to Beijing Tsingke Biotechnology Co., Ltd. (Nanjing, China) for sequencing and comparison. At the same time, we used EndoFree Mini Plasmid Kit II (Tiangen, Beijing, China) to extract the plasmid in the remaining bacterial solution for cell transfection according to its instructions. The successfully constructed vector was named pIGFBP5-O.

### 2.5. Synthesis of PAPPA2 Small Interfering RNA (siRNA)

The siRNA of *PAPPA2* is designed and synthesized by Shanghai GenePharma Co., Ltd. (Shanghai, China). The final sequence is shown in Table 3. We added 125 µL of RNase-Free ddH_2_O to the siRNA lyophilized powder according to its instructions to dilute it to a 20 µM stock solution, and store it at −20 °C for later use after dispensing to avoid repeated freezing and thawing.

### 2.6. Cell Transfection

The 4th generation DPCs with good growth status were inoculated uniformly in 6-well plates. When the cell confluence reached 50–80%, we used jetPRIME^®^ transfection reagent (Polyplus transfection, Illkirch, France) for DNA or siRNA transfection of DPCs referring to its instructions. Each transfection test group was set up as follows: pPAPPA2-O/pcDNA3.1(+), pIGFBP5-O/pcDNA3.1(+) and siRNA/NC. The transfection concentration is 1–3 μL/well for DNA and 10–60 nM for siRNA.

### 2.7. Cell Cycle Assay

When the cell confluence reached 80% after 36 h of transfection, this research used the Cell Cycle and Apoptosis Analysis Kit (Beyotime, Shanghai, China) to detect the changes in the ratio of the G1, S, and G2 stage cells of Hu sheep DPCs proliferation. After the staining was completed, the BD LSRFortessa flow cytometer (BD, Franklin Lakes, NJ, USA) was used to detect the red fluorescence produced by about 20,000 cells excited by the wavelength of 488 nm.

After completing flow cytometry, Modfit (Version: 3.1) (Verity Software House, Topsham, ME, USA) was used to analyze the data, and the proportion of cells in G1, S and G2 phases was calculated.

### 2.8. EdU and High-Content Image Acquisition and Analysis

5-Ethynyl-2′-Deoxyuridine (EdU) is an analogue of thymine, which can replace thymine to be incorporated into the replicating DNA molecule during cell proliferation. The rate of cell proliferation can be detected by detecting the ratio of EdU-positive cells to the total number of cells.

EdU test finished in this study was performed in 96-well plates using 4th generation DPCs of Hu sheep. The experiment was carried out according to the instruction of Cell-Light™ EdU Apollo 567 In Vitro Kit (Ribobio, Guangzhou, China) The general steps are as follows:

DPCs in logarithmic growth phase were inoculated and cultured in 1 × 10^4^ cells/well for 12 h until the cells adhered to the wall. Then we followed the cell transfection step mentioned in 1.1.6 but did not change the medium until 12 h after cell transfection. Technical replicates of 5 wells were set up for each group. After 48 h of cell transfection, the medium was removed and 100 μL of diluted EdU reagent (diluted in 1:1000 medium) was add to each well. Another 2 h of incubation was needed. When incubation finished, the well with PBS was washed for 5 min twice. Then we added 50 μL 4% paraformaldehyde cell fixation solution to each well and incubated for 30 min at room temperature. After the fixation, 2 mg/mL glycine was used to neutralize the aldehyde group. The next operation is washing with PBS once, then incubating with PBS containing 0.5% TritonX-100 on a shaker for 10 min and wash with PBS. Apollo staining solution was prepared according to the kit’s instructions and was added to the wells then incubated for 30 min in the dark. Again, incubate twice with PBS containing 0.5% TritonX-100 on a shaker and wash with methanol 5 min in addition PBS for 5 min. Hoechst333442 was provided in the kit for nuclear staining. Finally, we washed 3 times with PBS and added PBS to store at 4 °C. The last step is taking pictures.

The pictures that can reflect the results of the experiment were taken and analyzed by fluorescence micrographs of cells in each well using the Operetta CLS high-content cell imaging system (PerkinElmer, Santa Clara, CA, USA). The Apollo 567 dye uses 550 nm as the excitation light and the emission wavelength is 565 nm. Therefore, when taking pictures, we selected a similar dye Cy3’s channel for photos, also used a 20× objective lens. Each hole we selected a central square area including 5×7 equals to 35 fields of view. When all the pictures are collected, the system can analyze the graphic data by inputting the Hoechst333442 and EdU photos of the same field of view, screening and counting the cells of the two photos. Due to the low intensity of EdU staining, an additional step of screening brightness threshold is added as a necessary condition for EdU-positive cells to remove unnecessary interference layers. Finally, the number of EdU-positive cells was compared with the total number of cells to obtain the rate of EdU-positive cells reflecting the cell proliferation rate. All the pictures in one hole was considered as technique repeats and calculated to output the results.

### 2.9. Data Analysis

The statistical data involved in the above experiments were all sorted and calculated using Microsoft Excel (Version: 2013, Microsoft, CA, USA). The significance test of the experimental data was carried out by SPSS (Version: R25.0.0.1) (IBM, Armonk, NY, USA) software. The comparison of two sets of data was calculated by the t test to calculate the significance of the difference. One-way analysis of variance was used for the comparison between multiple sets of data to select the LSD as well as Dunnett’s T3 method and calculate the significance of the difference according to the homogeneity of the variance. All experiments were repeated at least thrice. When using GraphPad Prism 8 (GraphPad Software, San Diego, CA, USA) to draw statistics, statistics such as fluorescence quantification, cell cycle and other statistics appearing in the picture were expressed as mean ± standard error (X ± SEM). *p* < 0.05 indicates that the difference is significant, which is indicated by an asterisk (*) in the chart, *p* < 0.01 means that the difference is highly significant, which is represented by two asterisks (**) in the chart, *p* > 0.05 means that there is no significant difference between the two groups, which is represented by “ns” in the chart.

## 3. Results

### 3.1. Construction of PAPPA2 and IGFBP5 Eukaryotic Overexpression Vectors

**Figure 3:**
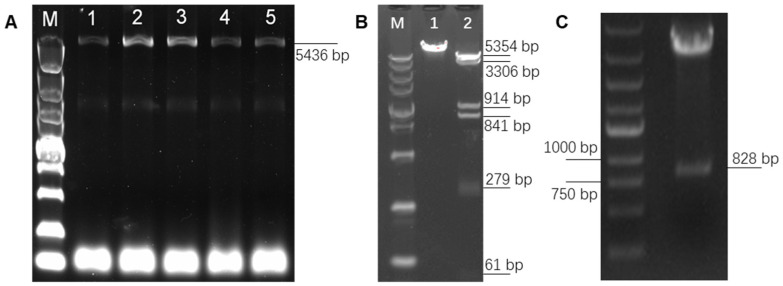
Construction of pPAPPA2-O and pIGFBP5-O. Note: (**A**): Full-length amplification of Hu sheep Pregnancy-Associated Plasma Protein A2 (*PAPPA2*) CDS, lane M is DL5000 marker, lanes 1–5 are PCR products with TM of 60, 59, 58, 57 and 56 °C, which shows that the optimum TM is 58–59 °C. (**B**): Single and double digestion verification of pPAPPA2-O. Lane M is DL5000 marker. Lane 1 is pPAPPA2-O digested by *Hin*d III for 30 min. Lane 2 is pPAPPA2-O digested by *Hin*d III and *Xho* I for 30 min. (**C**): Double digestion verification of pIGFBP5-O using *Bam*H I and *Eco*R I.

In order to overexpress *PAPPA2* in DPCs and explore its function, high-fidelity PCR primers were designed based on the 5382 bp CDS sequence of *PAPPA2* (XM_004013823.4) provided by NCBI (https://www.ncbi.nlm.nih.gov/ (accessed on 22 November 2020)), and a full-length 5436 bp target fragment was amplified by high-fidelity amplification using the reverse transcription product of freshly extracted DPCs RNA as a template (Figure 3A). The target fragment was purified by gel cutting and constructed into the pcDNA3.1(+) eukaryotic expression vector. The successfully constructed vector was verified by single digestion *(Xho* I) and double digestion (*Hin*d III and *Xho* I) (Figure 3B). In this figure, lane 1 was the result of single digestion and showed a single band, lane 2 was the result of double digestion, and since the *PAPPA2* mRNA sequence itself contains four *Hin*d III digestion sites inside, the result showed six bands, of which the 5354 bp band was pcDNA3.1(+) linear vector, and the remaining five bands were fragments of the *PAPPA2* CDS. Further sequencing verification showed that compared to the reference sequence (XM_004013823.4), seven inconsistencies were found in the presently obtained sequence, resulting in four synonymous mutations and three missense mutations (Table 4). By comparing the sequence of sheep PAPPA2 recorded on UniProt (https://www.uniprot.org/ (accessed on 22 November 2020)), it was found that all protein mutation sites (N-terminal positions 391, 1231, 1690) translated by the full-length CDS sequence of Hu sheep *PAPPA2* amplified in this study could be matched with W5PZE1_SHEEP (UniProt accession number: W5PZE1), but also some loci (N-terminal positions 1442, 1457, 1640) that were consistent with the NCBI reference sequence showed differences with W5PZE1_SHEEP (Table 5). Together with the consistent sequences of multiple vectors constructed in this study, we deduced that no mutations were introduced during the construction (mainly during amplification and ligation). Thus, the sequence we obtained is wild-type *PAPPA2*. The successfully constructed vector was named pPAPPA2-O.

To elucidate the interrelationship between *IGFBP5* and *PAPPA2*, the eukaryotic overexpression vector of *IGFBP5* (pIGFBP5-O) was constructed in this study. The full length of the CDS region of *IGFBP5* was 816 bp, and the Kozak sequence was added at the 5′ end of the CDS region, and the final digested product was 828 bp (Figure 3C). The inserts in the synthesized pIGFBP5-O were sequenced and compared with the *IGFBP5* mRNA sequence (NM_001129733.1), and no base mismatches were found.

### 3.2. PAPPA2 Promotes the Proliferation of Hu Sheep DPCs

**Figure 4:**
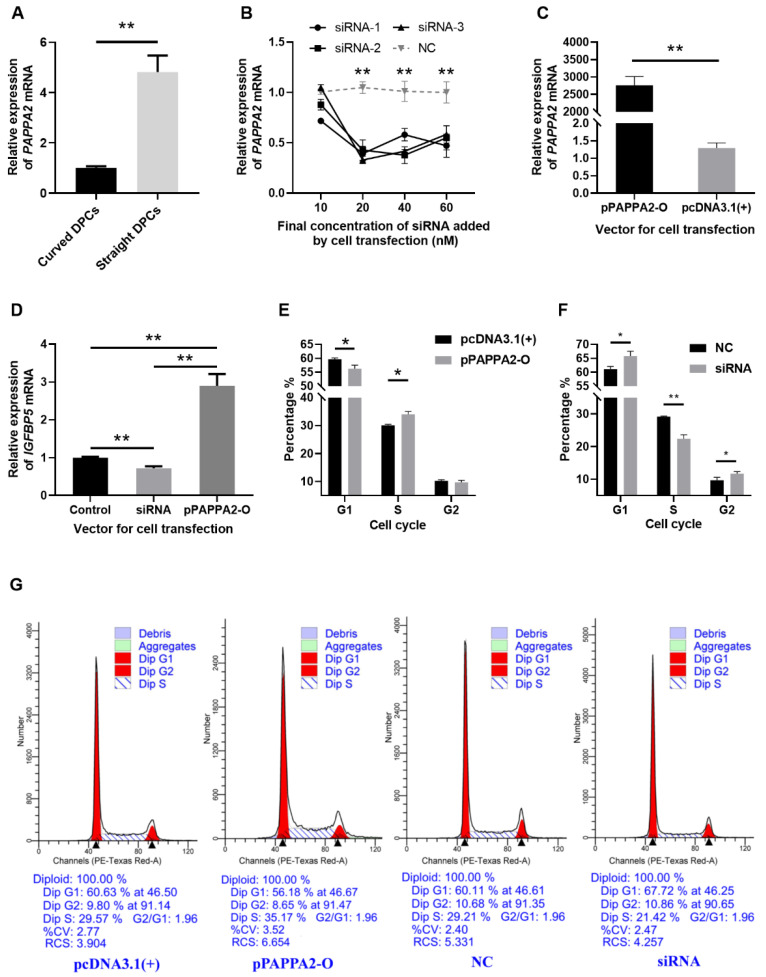
*PAPPA2* was involved in the regulation of DPCs proliferation in Hu sheep. Note: (**A**): Relative expression of *PAPPA2* mRNA in straight and curved dermal papilla cells (DPCs). (**B**): Relative expression of *PAPPA2* after transfection with different concentrations of *PAPPA2* siRNA-1/2/3 (with NC as control). (**C**): Relative expression of *PAPPA2* after pPAPPA2-O transfection (with pcDNA3.1(+) as control). (**D**): *PAPPA2* regulate the mRNA expression of insulin-like growth factor binding protein-5 (*IGFBP5*). (**E**,**F**) show the cell cycle statistics of DPCs after *PAPPA2* overexpression (transfected with pPAPPA2-O) and interference (transfected with siRNA-1/2/3). The Transfections of pcDNA3.1(+) and NC were used as controls for overexpression and interference, respectively. (**G**): The results of cell cycle analysis by ModFit3.1. “*” indicates significant difference (*p* < 0.05) and “**” highly significant difference (*p* < 0.01).

Firstly, to verify that *PAPPA2* is indeed differentially expressed between DPCs from curved and straight hair in Hu sheep, RNA was extracted from the third-generation DPCs of curved and straight hair, and we used RT-qPCR, with *GAPDH* as an internal reference, for relative quantification of *PAPPA2* mRNA expression in DPCs of curved and straight hair (Figure 4A). The results revealed that *PAPPA2* expression in straight DPCs was significantly higher than that in curved DPCs (*p* < 0.01).

To verify the effectiveness of the siRNA and overexpression vector of *PAPPA2*, *PAPPA2* siRNA-1/2/3 and pPAPPA2-O were transfected into the 4th generation DPCs cultured in 12-well plates. The experimental groupings were set as follows: In order to test the effect and optimal concentration of these three siRNA, DPCs were transfected with siRNA-1, siRNA-2 and siRNA-3 (test group) and NC (control group), each with 4 concentrations: 10/20/40/60 nM, and three replicates for each condition. To validate the effect of *PAPPA2* overexpression vector, 2 μg pPAPPA2-O (test group) and pcDNA3.1(+) (control group) were transfected into DPCs, with three replicates for each condition. The expression of *PAPPA2* mRNA was quantified relatively using *GAPDH* as the internal reference gene. Transfecting *PAPPA2* siRNA-1/2/3 at a final concentration ≥ 20 nM in DPCs of Hu sheep reduced the expression of *PAPPA2* mRNA very significantly (*p* < 0.01), indicating a stable inhibition effect (Figure 4B), thus we determined that subsequent experiments transfecting one replicate well each with siRNA-1/2/3 at a final concentration of 40 nM (as three biological replicates) were used to inhibit *PAPPA2* expression. In addition, transfecting pPAPPA2-O in DPCs of Hu sheep was able to significantly increase their *PAPPA2* mRNA expression (*p* < 0.01) (Figure 4C).

To further study whether *PAPPA2* also plays a role through regulating *IGFBP5* in DPCs of Hu sheep as in humans, with *GAPDH* as an internal reference, the same cDNA as the above experiments (transfection of 40 nM siRNA-1/2/3 and 2 μg pPAPPA2-O) was used for the relative quantification of *IGFBP5* mRNA expression in DPCs. The data were processed with pcDNA3.1(+) and NC as controls. As shown from Figure 4D, compared with the control group, overexpression of *PAPPA2* significantly increased the mRNA expression of *IGFBP5* in DPCs (*p* < 0.01). In contrast, the mRNA expression of *IGFBP5* was significantly lower in the interfered group than the control group (*p* < 0.01). This result suggests that *PAPPA2* is positively correlated with the expression of *IGFBP5* mRNA in DPCs of Hu sheep. This may be indirectly caused by the degradation of IGFBP5 by PAPPA2.

To characterize the function of *PAPPA2* in Hu sheep’s DPCs, the 4th generation straight were treated with the optimal transfection conditions determined in the above experiments, and cell cycle assays were performed 36 h after completion of cell transfection when cell confluency exceeded 80%. Overexpression of *PAPPA2* significantly increased the proportion of S phase in DPCs compared with the control group (*p* < 0.05) (Figure 4E). In contrast, the results of the interference test showed that not only the proportion of S phase in DPCs was significantly reduced compared with the control group (*p* < 0.01), but also the proportion of the G2 phase was significantly increased (*p* < 0.05) (Figure 4F). The above results indicated that overexpression of *PAPPA2* had a certain proliferation-promoting effect on DPCs, while interference with *PAPPA2* led to a decrease in the proliferation rate of DPCs and a certain degree of G2/M arrest.

### 3.3. PAPPA2 Can Restore the Proliferation of DPCs from Inhibition by IGFBP5

**Figure 5:**
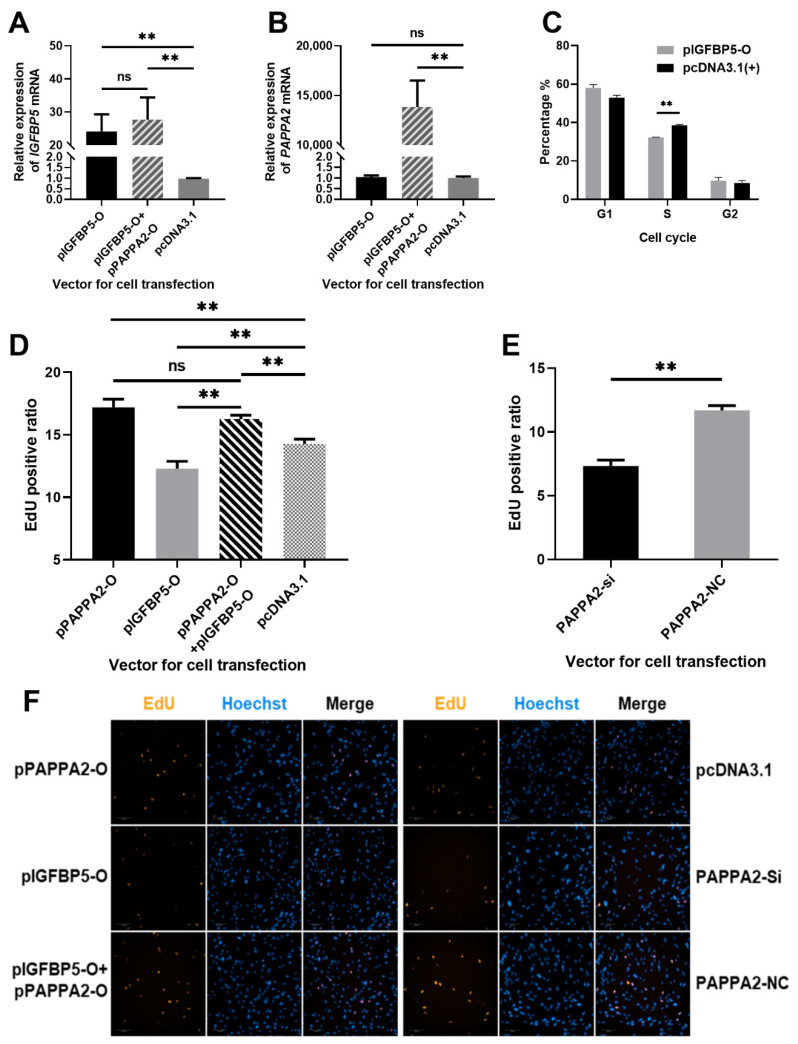
Effect of *PAPPA2* and *IGFBP5* on the proliferation of DPCs. Note: (**A**): Relative expression of *IGFBP5* after transfection of pIGFBP5-O, pIGFBP5-O+pPAPPA2-O and pcDNA3.1 in DPCs. (**B**): Relative expression of *PAPPA2* after transfection of pIGFBP5-O, pIGFBP5-O+pPAPPA2-O and pcDNA3.1 in DPCs, respectively. (**C**): Effect of *IGFBP5* on the cycle of DPCs. (**D**): Proportion of EdU-positive cells after transfection with pIGFBP5-O, pPAPPA2-O, pIGFBP5-O+pPAPPA2-O and pcDNA3.1 in DPCs, respectively. (**E**): Proportion of EdU-positive cells after transfection with siRNA-2 and siRNA-NC of *PAPPA2* in DPCs, respectively. (**F**): Fluorescence photos of EdU staining results in each group (200×). In this figure, “**” highly significant difference (*p* < 0.01) and “ns” indicates non-significant difference.

To verify the effect of pIGFBP5-O and pPAPPA2-O co-transfection, pPAPPA2-O and pIGFBP5-O were transfected into 4th generation DPCs respectively. The experimental groupings were set as follows: DPCs transfected with 2 μg pIGFBP5-O as *IGFBP5* overexpression test group; DPCs transfected with 1.5 μg pIGFBP5-O and 1.5 μg pPAPPA2-O as *IGFBP5* and *PAPPA2* co-overexpression test group; DPCs transfected with 2 μg pcDNA3.1(+) as a control group. All subgroups were set up with 3 replicates. Cell total RNA was extracted 24 h after transfection, and the expression levels of *IGFBP5* and *PAPPA2* mRNA were detected by RT-qPCR using *GAPDH* as an internal reference gene. The expression levels of *IGFBP5* in both *IGFBP5* overexpression group and the co-overexpression group were significantly increased compared to the control group (*p* < 0.01), and the difference between these two groups was not significant (*p* > 0.05) (Figure 5A). Furthermore, the relative expression level of *PAPPA2* was only significantly increased in the co-overexpression group compared with the control group (*p* < 0.01), and the expression of *PAPPA2* was no change after overexpression of *IGFBP5* (Figure 5B). Thus, this study successfully overexpressed *IGFBP5* and *PAPPA2* simultaneously in DPCs, suggesting that the method can be used for the next experiments.

In order to study the function of *IGFBP5* in Hu sheep DPCs, pIGFBP5-O was transfected into the 4th generation straight DPCs, and cell cycle was assayed 36 h after completion of cell transfection when cell confluency exceeded 80%. The proportion of cells in S phase in the *IGFBP5* overexpression group was highly significantly lower than that in the control group (*p* < 0.01) (Figure 5C). This result indicated that *IGFBP5* was able to inhibit the proliferation of DPCs, especially arrest cells in G1/S checkpoint.

To further clarify the effect of *PAPPA2* and *IGFBP5* on the proliferation of DPCs, the DNA replication of DPCs under different transfection conditions was examined in this study using the EdU Cell Proliferation Kit. The specific groups were as follows: DPCs transfected with 100 ng/well pPAPPA2-O as *PAPPA2* overexpression group; DPCs transfected with 100 ng/well pIGFBP5-O as *IGFBP5* overexpression group; DPCs transfected with both 75 ng/well pPAPPA2-O and 75 ng/well pIGFBP5-O as *IGFBP5* & *PAPPA2* co-overexpression group; DPCs transfected with 100 ng/well pcDNA3.1(+) as the control group of the above 3 groups; DPCs transfected with siRNA-1/2/3 at a final concentration of 40 nM as the *PAPPA2* inhibition group; DPCs transfected with siRNA-NC at a final concentration of 40 nM as the control group. All subgroups were set up with 5 replicates. A total of 1050 images (200× magnifications) were obtained after EdU stain using the high-content cell imaging system (Figure 5D–F). The proliferation rate of DPCs in the *PAPPA2* overexpression group was significantly increased compared to the control group (*p* < 0.01), whereas the cell proliferation rate in the *IGFBP5* overexpression group was significantly decreased (*p* < 0.01). Compared to the group only overexpressed *IGFBP5*, co-overexpression of *IGFBP5* and *PAPPA2* resulted in a significant increase in the proliferation rate of DPCs (*p* < 0.01), and there was no significant difference in cell proliferation rate between the *PAPPA2* overexpression group and *IGFBP5* & *PAPPA2* co-overexpression group (*p* > 0.05) (Figure 5D). In the experiment of interfering with the expression of *PAPPA2* in DPCs alone, we found that cell proliferation decreased significantly as in the experiment of overexpression of *IGFBP5* (*p* < 0.01) (Figure 5E).

Both cell cycle and EdU assays revealed that overexpression of *PAPPA2* in Hu sheep DPCs promoted their proliferation and interference of *PAPPA2* inhibited cell proliferation. Overexpression of *IGFBP5* alone in DPCs led to a decrease in cell proliferation, but overexpression of *IGFBP5* together with *PAPPA2* restored the proliferation rate of DPCs inhibited by *IGFBP5*, even comparable to that of *PAPPA2* alone. Thus, *PAPPA2* and *IGFBP5* can regulate the proliferation of Hu sheep DPCs, and there is a strong correlation between these two.

## 4. Discussion

*PAPPA2* is a gene that has received less scholarly attention, and studies related to it have focused on the areas of pregnancy risk [24], child development [19], and skeletal development [20]. It was not clear that *PAPPA2* has an association with hair growth. In 2001 Overgaard et al. [18] hypothesized the existence of another similar exocrine protein, PAPPA2, based on an identified gene: *PAPPA*, and successfully expressed it in human embryonic kidney 293T cells by constructing its overexpression vector. This report confirms the presence of *PAPPA2* for the first time. Glu-734 of PAPPA2, similar to PAPPA’s specific hydrolysis of IGFBP4, has hydrolytic activity on IGFBP5. In human, *PAPPA2* is able to specifically cleave IGFBP5 at its site between Ser-143 and Lys-144, while PAPPA2 has a mild enzymatic cleavage effect on IGFBP3. *PAPPA2* can play a role in normal development of children, and several rare dysfunctional mutations of *PAPPA2,* resulting in short stature and elevation of *IGF1* levels as well as skeletal dysplasia, were reported in 2016. These children treated with recombinant IGF1 recovered to some degree [25,26].

The IGFBP5 in sheep consists of 271 amino acids, and the UniProt annotation was retrieved for its two structural domains, the amino (N) terminus (amino acids 22–102) and the carboxyl (C) terminus (amino acids 188–262). A study conducted by Andress et al. [27] in 1992 found that a 23 kDa IGFBP5 (residue) purified from human osteoblast-like cells was able to stimulate cellular proliferation in the absence of IGF synergy, and interestingly, The size of this IGFBP5 was identical to that of IGFBP5 fragment cleaved by PAPPA2 [18]. A carboxyl-truncated IGFBP5 (containing only 169 amino acids at the N-terminal end) was synthesized and co-incubated with human osteoblast-like cells by Andress in another study and he found that this carboxyl-truncated IGFBP5 was able to bind to a receptor of 420 kDa in size on the surface of the cell membrane [28]. Further studies revealed that this binding was able to phosphorylate the receptor [29]. Additional studies have shown that IGFBP5 can be transported by cells from the extracellular to the nucleus, which in turn seems to suggest other pathways for IGFBP5 to exert its role [30]. Thus, intact IGFBP5 can effectively bind IGF and inhibit cell proliferation, however, when PAPPA2 hydrolyzes IGFBP5 (with or without binding IGF) into two separate fragments, not only IGF will be released to promote cell proliferation, but also the IGFBP5 residues generated by hydrolysis can further stimulate cell proliferation, and this mechanism provides a possibility for regulation of local cell proliferation in animals.

In this study, we first identified that the expression of *PAPPA2* was higher in Hu sheep straight DPCs than in curved. Then, overexpression of *PAPPA2* in Hu sheep DPCs showed a significant increase in the proliferation rate of DPCs, which is consistent with the theory that *PAPPA2* is involved in regulating cellular utilization of IGF, thereby promoting cell growth. [19,31,32]. Further examination about the expression of *IGFBP5*, a gene downstream of *PAPPA2*, revealed that overexpression of *PAPPA2* resulted in elevated mRNA expression of *IGFBP5*, which may lead to more IGFBP5 being hydrolyzed, causing it to stimulate cell mitosis from another pathway. Therefore, in this study, we attempted to overexpress *IGFBP5* in DPCs to characterize its effect on the proliferation of DPCs and the interaction between *IGFBP5* and *PAPPA2*. In contrast to overexpression of *PAPPA2*, overexpression of *IGFBP5* alone in Hu sheep’s DPCs reduced the proliferative capacity of DPCs, which is consistent with the inhibitory effect of *IGFBP5* on cell proliferation found by other investigators in mouse embryonic fibroblasts [33]. The EdU results showed that overexpression of *IGFBP5* together with *PAPPA2* was able to revert the proliferative ability of DPCs back to the same proliferative capacity of overexpression of *PAPPA2* alone, which indicate that the relationship between *PAPPA2* and *IGFBP5* is not antagonistic but regulatory and regulated, in other words, *PAPPA2* completely regulates the intercellular *IGFBP5* signaling. In contrast, *IGFBP5* did not seem to have a regulatory effect on *PAPPA2*, because overexpression of *IGFBP5* did not alter the expression of *PAPPA2*. The above results are consistent with the known mechanism at work of *PAPPA2* that has been shown to rapidly and completely hydrolyze IGFBP5, suggesting that *PAPPA2* is able to independently regulate the content of *IGFBP5* around cellular. The results of all *PAPPA2* interference assays were opposite to overexpression, indicating that the results in this study are sufficiently plausible. The pattern formation in Hu sheep lambs was related to the length and time of fetal hair growth along the skin folds during the embryonic period. When the hair is short, it will form a beautiful wave-like pattern, while the overgrown fetal hair will gradually change this pattern to straight hair [2]. Therefore, slightly reducing the growth rate of fetal hair is a potential method to turn more Hu sheep lambs with medium curved, little curved and even straight pattern phenotype into lambs with an extremely curved pattern. Another study showed a significant correlation between the number of DPCs in individual hair follicles and the type of mouse hair. The gradual ablation of DPCs over multiple hair follicle cycles in test rats observed the growth of zigzag hairs in hair follicles that originally grew straight hairs [34]. Moreover, the hair follicle diameter of the extremely curved type was significantly smaller than that of the little curved type in different patterns of Hu sheep lambs [35], which seems to indirectly prove that hair thinning due to the number of DPCs can affect the pattern phenotype of Hu sheep lambs. Furthermore, *PAPPA2*, the differential gene between extremely curved and straight DPCs validated in this study, was proved to be a sufficient regulator of the DPCs’ proliferation. Therefore, this study makes the following inferences based on existing studies. The differential expression of *PAPPA2* in DPCs causes different degrees of hydrolysis of IGFBP5. In DPCs with high expression of *PAPPA2*, the massive hydrolysis of IGFBP5 leads to increased cellular utilization of IGF, which individually promotes the proliferation of DPCs in Hu sheep. A greater number of DPCs leads to thicker and longer hairs, which could result in the loss of the original wavy pattern during fetal hair growth and eventually to a straight hair phenotype. In contrast, low expression of *PAPPA2* reduced the thickness and length of fetal hair through the same pathway, which allows lambs to retain their curved hair at birth and form a wavy pattern phenotype.

## 5. Conclusions

Overall, this study identified a gene that is involved in the regulation of Hu sheep lambskin pattern: *PAPPA2*, differentially expression of which regulates proliferative rates in DPCs, and participate in the regulation of the type of Hu sheep lambskin pattern eventually.

## Figures and Tables

**Table 1:** Primers information.

Genes	Accession No.	Sequences (5′→3′) (F: Forward R: Reverse)	Product Length (bp)
*PAPPA2*	XM_004013823.4	F: CAGGGGCTCCATTCAACAAC	124
		R: CTCTGGCTCCACTGCTGATAC	
*IGFBP5*	NM_001129733.1	F: CTGTGACCGCAAAGGGTTCT	135
		R: CACTGAAAGTCCCCGTCCAC	
*GAPDH*	NM_001190390.1	F: TCTCAAGGGCATTCTAGGCTAC	151
		R: GCCGAATTCATTGTCGTACCAG	

**Table 2:** Enzyme digestion system.

Name	Volume or Mass
pcDNA3.1(+)	2 µg
10× QuickCut Green Buffer	4 µL
QuickCut™ *Hin*d III	2 µL
QuickCut™ *Xho* I	2 µL
RNase Free ddH_2_O	Up to 40 µL

**Table 3:** Sequences of *PAPPA2* siRNA and NC.

Name	Binding Sites	Sequences (5′→3′)
siRNA-1	2149	sense: CCUCAACCCAGCCUAUUAUTT
		antisense: AUAAUAGGCUGGGUUGAGGTT
siRNA-2	3965	sense: CCGCUGGUUAUCAACAUAATT
		antisense: UUAUGUUGAUAACCAGCGGTT
siRNA-3	4693	sense: GGGAAGCACAGAGAAUAAATT
		antisense: UUUAUUCUCUGUGCUUCCCTT
NC	-	sense: UUCUCCGAACGUGUCACGUTT
		antisense: ACGUGACACGUUCGGAGAATT

**Table 4:** Mutation locus of *PAPPA2* CDS in Hu sheep.

Name	Position (bp)	Reference	Mutant	Mutation Type
SNP1	1116	C	T	Missense mutation
SNP2	1171	C	G	Same Sense mutation
SNP3	1272	G	T	Same Sense mutation
SNP4	3489	G	A	Same Sense mutation
SNP5	3692	G	C	Missense mutation
SNP6	4704	A	G	Same Sense mutation
SNP7	5089	G	A	Missense mutation

Note: The reference sequence is XM_004013823.4 (NCBI), and the mutation site starts with the first base (A) in the CDS region.

**Table 5:** Amino acid mutation (AAM) of PAPPA2 in Hu sheep.

Name	Position (N to C)	Reference 1	Reference 2	Hu Sheep
AAM1	391	H	**D**	**D**
AAM2	1231	R	**P**	**P**
AAM3	1442	**M**	L	**M**
AAM4	1457	**R**	G	**R**
AAM5	1640	**Q**	R	**Q**
AAM6	1697	V	**I**	**I**

Note: The reference 1 is XP_004013872.2 (NCBI) and the reference 2 is W5PZE1_SHEEP (UniProt). The boldface of amino acid indicates the same amino acid as the Hu sheep’s PAPPA2.

## Data Availability

Data are available from the corresponding author upon reasonable request.
